# Supplementary material for: PERK Signaling Regulates Extracellular Proteostasis of an Amyloidogenic Protein During Endoplasmic Reticulum Stress
Source: Sci Rep. 2019 Jan 23;9:410. doi: 10.1038/s41598-018-37207-0 (PMC6344643; doi:10.1038/s41598-018-37207-0)

Supplement to:

PERK Signaling Regulates Extracellular Proteostasis of an Amyloidogenic Protein During Endoplasmic Reticulum Stress

Isabelle C. Romine and R. Luke Wiseman*

Department of Molecular Medicine, The Scripps Research Institute, La Jolla, CA 92037

*To whom correspondences should be addressed:
R. Luke Wiseman
Department of Molecular Medicine
The Scripps Research Institute
10550 N. Torrey Pines Rd. MEM 220
La Jolla, CA 92037
Email: wiseman@scripps.edu
Phone: (858) 784-8820

Running Title: PERK regulates extracellular proteostasis

Supplemental Information Table of Contents

Page	Title	Description
1	Cover Page	Description of Supplemental Content
2	Supplemental Figure 1	Relevant to Figure 1
3	Supplemental Figure 2	Relevant to Figure 2
4	Supplemental Figure 3	Relevant to Figure 3
5	Supplemental Figure 4	Relevant to Figure 4
6	Whole gels for Figure 1A	Raw data for Figure 1A
7	Whole gels for Figure 2E	Raw data for Figure 2E
8	Whole gels for Figure 3C	Raw data for Figure 3C
9	Whole gels for Figure S1A	Raw data for Figure S1A
10	Whole gels for Figure S1F	Raw data for Figure S1F
11	Whole gels for Figure S3	Raw data for Figure S3
12	Whole gels for Figure S4A	Raw data for Figure S4A
13	Whole gels for Figure S4B	Raw data for Figure S4B
14	Whole gels for Figure S4C	Raw data for Figure S4C

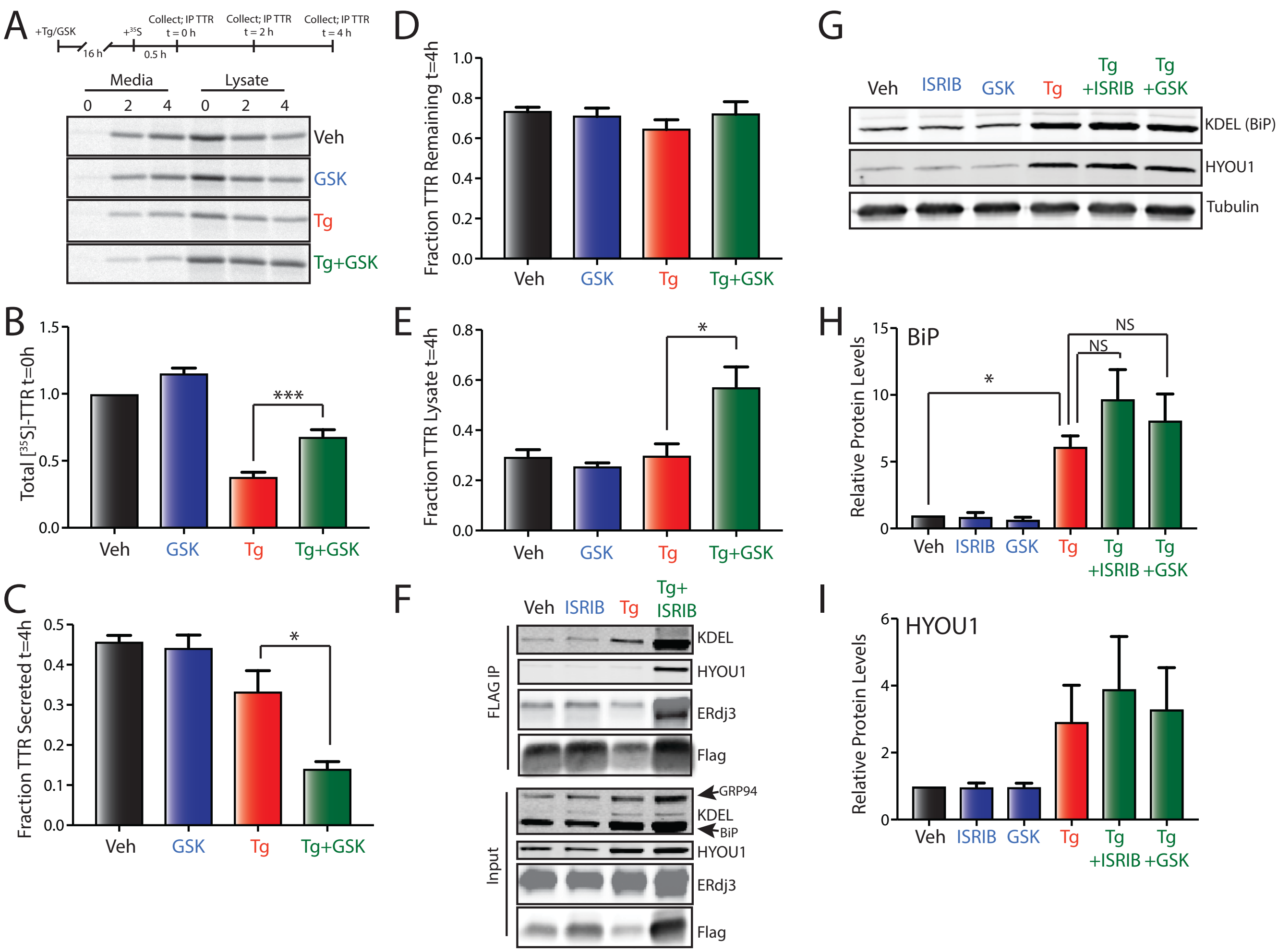

Supplemental Figure 1 (Relevant to Figure 1)

- **A.** Representative autoradiogram of [^{35}S]-labeled ^{FT}TTR^{A25T} in lysates and media collected from HEK293T cells pretreated for 16 h with thapsigargin (Tg; 500 nM) and/or GSK (300 nM). The protocol for the experiment is shown above.
- **B.** Graph showing the total amount of [^{35}S]-labeled ^{FT}TTR^{A25T} at t = 0 h in HEK293T cells pretreated for 16 h with thapsigargin (Tg; 500 nM) and/or GSK (300 nM). Data are shown normalized to vehicle-treated cells. Error bars show SEM for n=3 independent experiments. A representative autoradiogram is shown in **Fig. S1A**.
- **C.** Graph showing the fraction [^{35}S]-labeled ^{FT}TTR^{A25T} secreted at t = 4 h in HEK293T cells pretreated for 16 h with thapsigargin (Tg; 500 nM) and/or GSK (300 nM). Fraction secreted was calculated as described in **Materials and Methods**. Error bars show SEM for n=3 independent experiments. A representative autoradiogram is shown in **Fig. S1A**.
- **D.** Graph showing the fraction [^{35}S]-labeled ^{FT}TTR^{A25T} remaining at t = 4 h in HEK293T cells pretreated for 16 h with thapsigargin (Tg; 500 nM) and/or GSK (300 nM). Fraction remaining was calculated as described in **Materials and Methods**. Error bars show SEM for n=3 independent experiments. A representative autoradiogram is shown in **Fig. S1A**.
- **E.** Graph showing the fraction [^{35}S]-labeled ^{FT}TTR^{A25T} in lysates at t = 4 h in HEK293T cells pretreated for 16 h with thapsigargin (Tg; 500 nM) and/or GSK (300 nM). Fraction lysate was calculated as described in **Materials and Methods**. Error bars show SEM for n=3 independent experiments. A representative autoradiogram is shown in **Fig. S1A**.
- **F.** Representative immunoblot showing anti-FLAG IPs and inputs from lysates prepared from HEK293T cells expressing ^{FT}TTR^{A25T} and pretreated for 16 h with Tg (500 nM) and/or ISRIB (200 nM)
  *indicates $p<0.05$; **indicates $p<0.01$ for a paired two-tailed t-test.
- **G.** Representative immunoblot of lysates prepared from HEK293T cells pretreated for 16 hours with Tg (500 nM), ISRIB (200 nM), and/or GSK (300 nM), as indicated.

H. Graph showing quantifications for BiP from immunoblots as shown in **Fig. 1G**. Data are shown normalized to vehicle-treated cells. Error bars show n=3. *indicates p<0.05.

I. Graph showing quantifications for HYOU1 from immunoblots as shown in **Fig. 1G**. Data are shown normalized to vehicle-treated cells. Error bars show n=3. *indicates p<0.05.

A

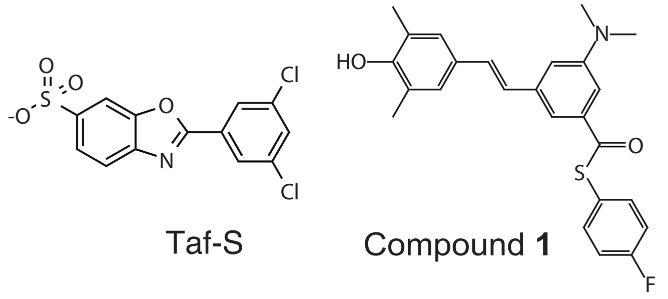

B

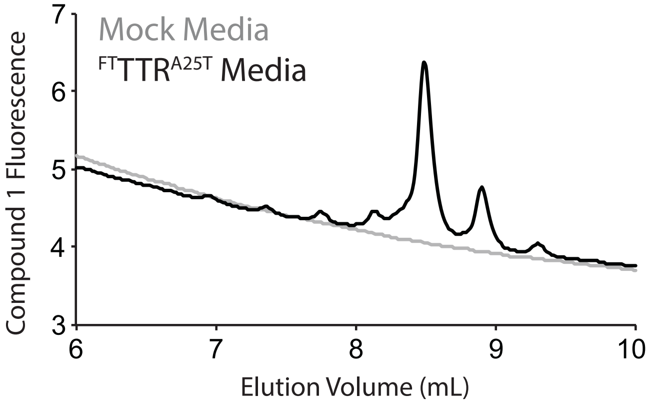

C

	DMSO	ISRIB	Tg	Tg + ISRIB
Parent	11.9 %	10.9 %	12.5 %	23.9 %
+80 (+SO_3)	33.5 %	29.8 %	26.8%	26.2 %
+120 (+Cys)	21.3 %	20.4 %	21.2 %	28.5 %
+160 (+2 SO_3)	5.4 %	3.3 %	3.5 %	ND
+200 (+SO_3 +Cys)	33.4 %	35.7 %	27.7 %	21.4 %
Other	ND	3.3 %	8.2 %	ND

Supplemental Figure 2 (Relevant to Figure 2)

A. Structures of tafamidis-sulfonate (Taf-S) and the fluorogenic TTR ligand compound **1.**

B. Chromatogram of conditioned media prepared in the presence of Taf-S (10 µM) on mock-transfected HEK293T cells or HEK293T cells expressing $^{FT}TTR^{A25T}$. $^{FT}TTR^{A25T}$ tetramers were separated on anion exchange chromatography using the UPLC system and visualized by compound **1** fluorescence.

C. Table showing the relative populations of unmodified and posttranslationally modified $^{FT}TTR^{A25T}$ in conditioned media prepared on HEK293T cells treated for 16 h with Tg (500 nM) and/or ISRIB (200 nM). Please see ***Materials and Methods*** for further description of this experiment.

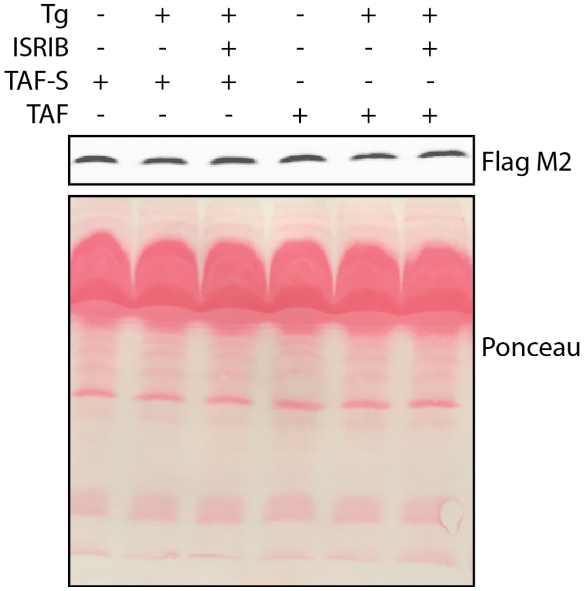

Supplemental Figure 3 (Relevant to Figure 3)

Representative immunoblot of $^{FT}TTR^{A25T}$ in conditioned media prepared in the presence of Taf-S (10 µM) or Taf (10 µM) on HEK293T cells treated for 16 h with Tg (500 nM) and/or ISRIB (200 nM). A ponceau stain of the immunoblot is shown as a control.

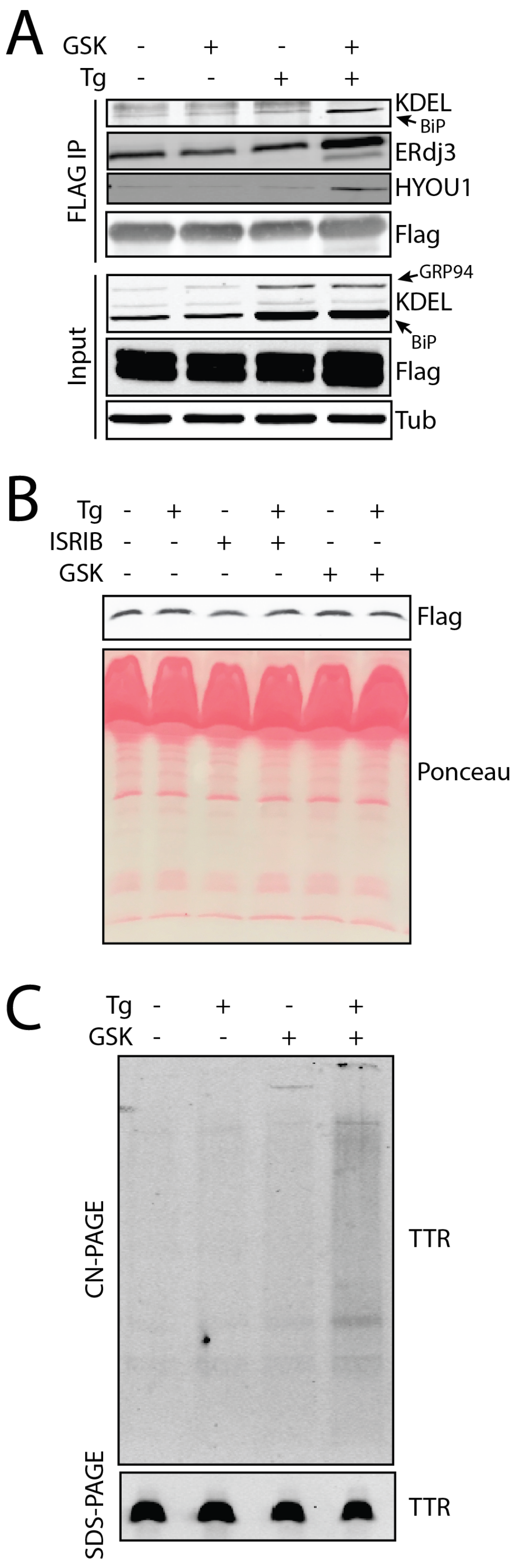

Supplemental Figure 4 (Relevant to Figure 4).

- **A.** Representative immunoblot showing anti-FLAG IPs and inputs from lysates prepared from HEK293Tcells expressing $^{FT}TTR^{WT}$ and pretreated for 16 h with Tg (500 nM) and/or GSK (300 nM).
- **B.** Representative immunoblot of $^{FT}TTR^{WT}$ in conditioned media prepared in the presence of Taf-S (10 μM) on HEK293T cells treated for 16 h with Tg (500 nM), ISRIB (200 nM), or GSK (300 nM). A ponceau stain of the immunoblot is shown as a control.
- **C.** Representative CN-PAGE/immunoblot of $^{FT}TTR^{WT}$ aggregates in conditioned media prepared in the presence of Taf-S (10 μM) on HEK293T cells treated for 16 h with Tg (500 nM) and/or GSK (300 nM). A SDS-PAGE/immunoblot showing total $^{FT}TTR^{WT}$ is shown as a control.

Figure 1A Raw Data (Whole Gel Exposure)

Vehicle

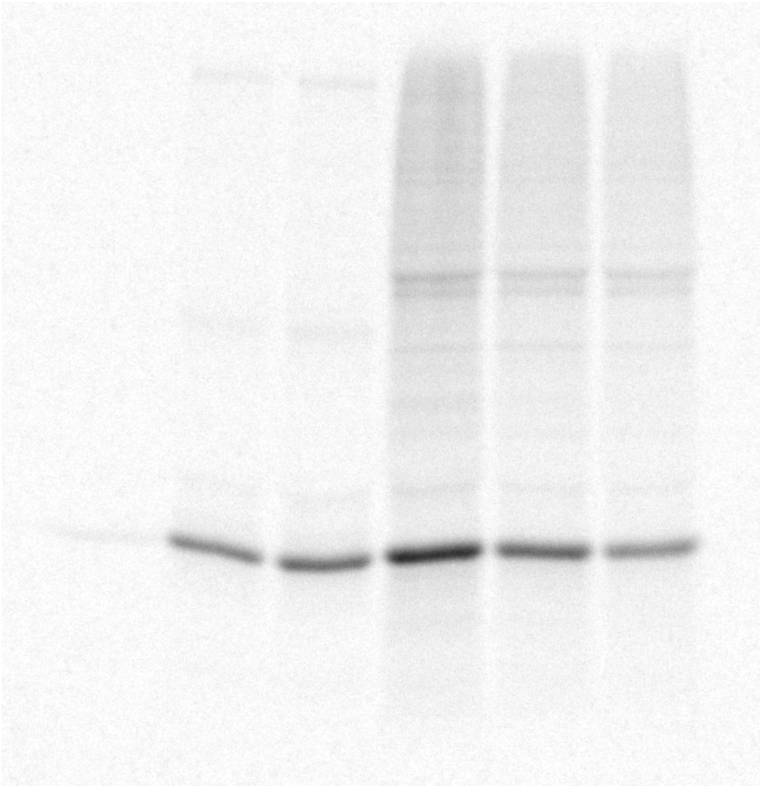

Thapsigargin

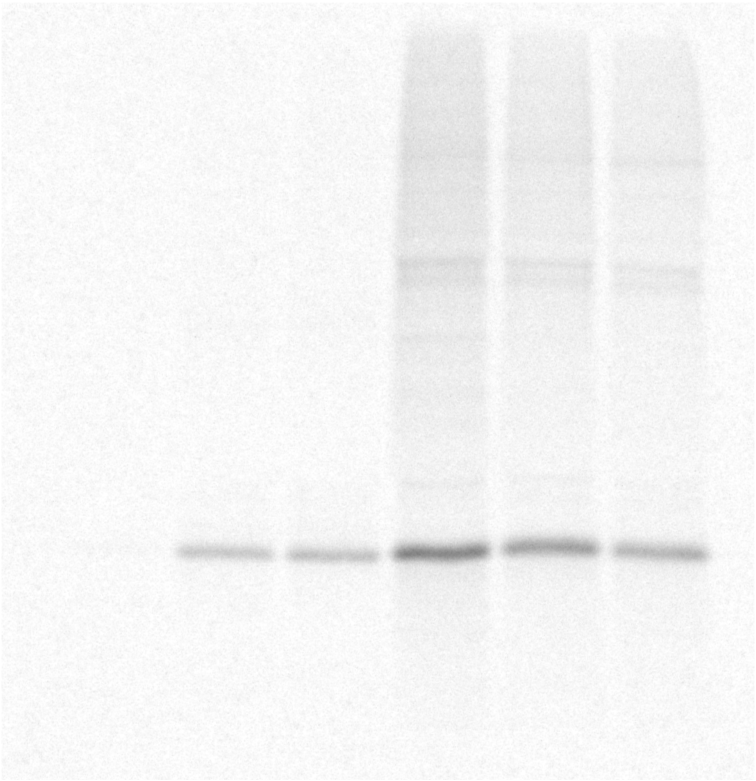

ISRIB

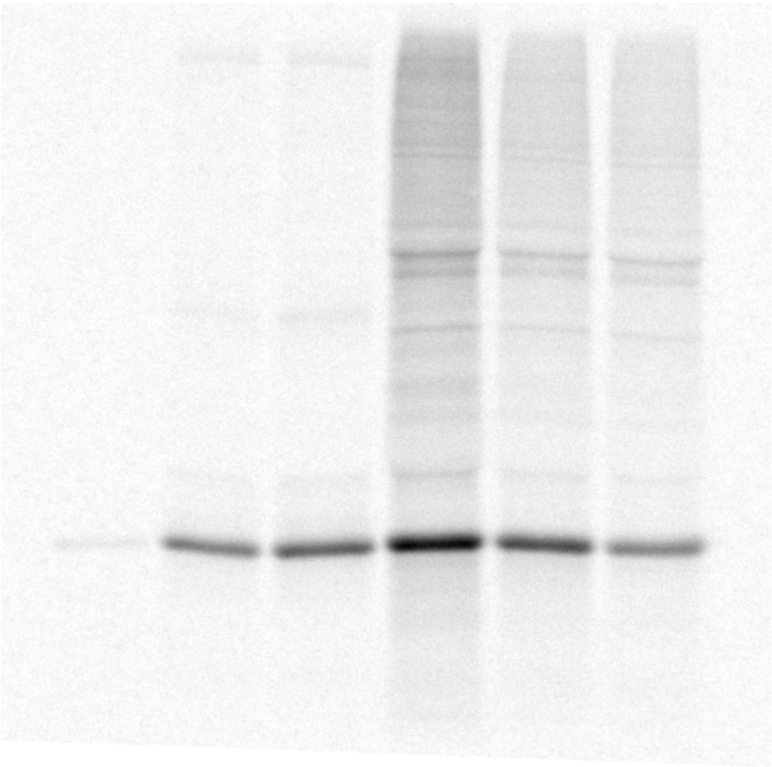

Thapsigargin and ISRIB

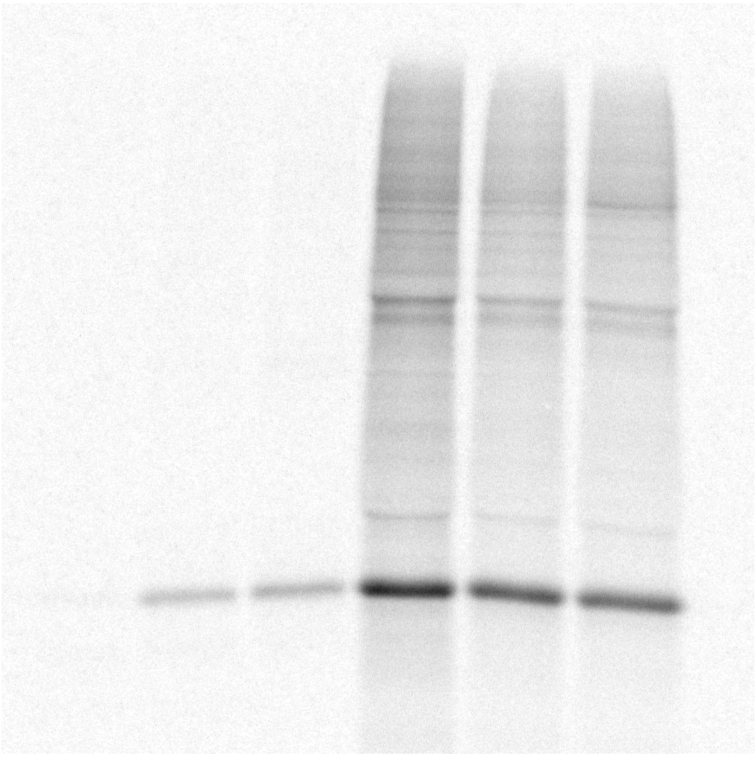

Figure 2E Raw Data (Whole Gels)

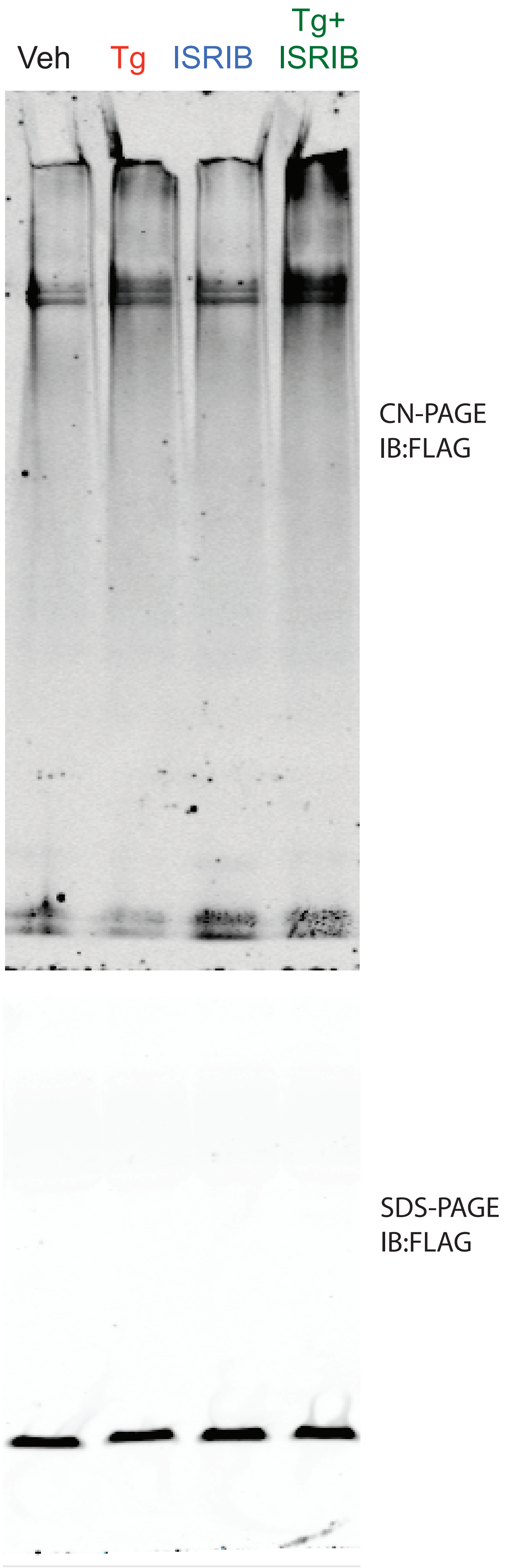

Figure 3C Raw Data (Whole Gels)

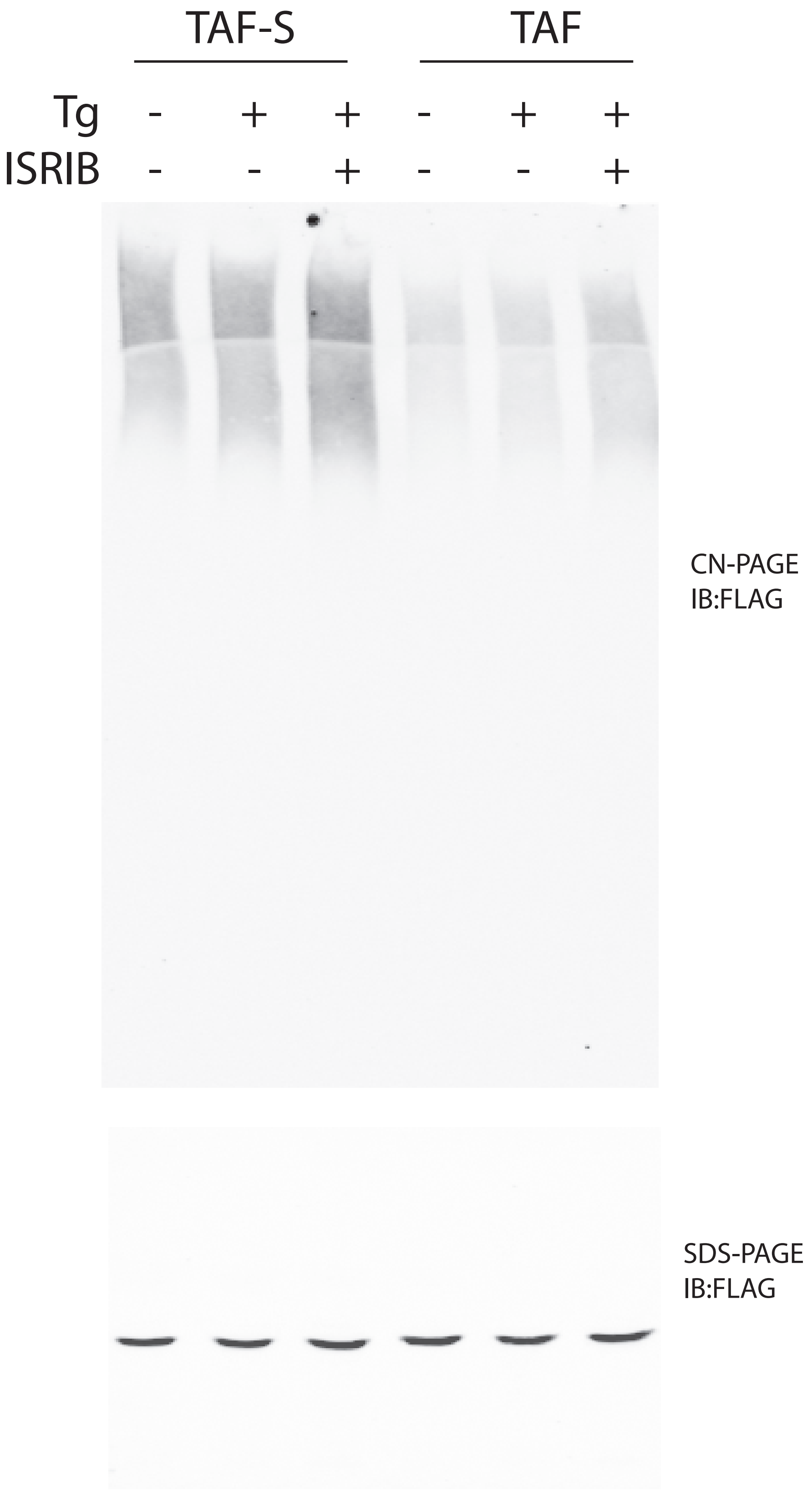

Figure S1A Raw Data (Whole Gel Exposure)

Vehicle

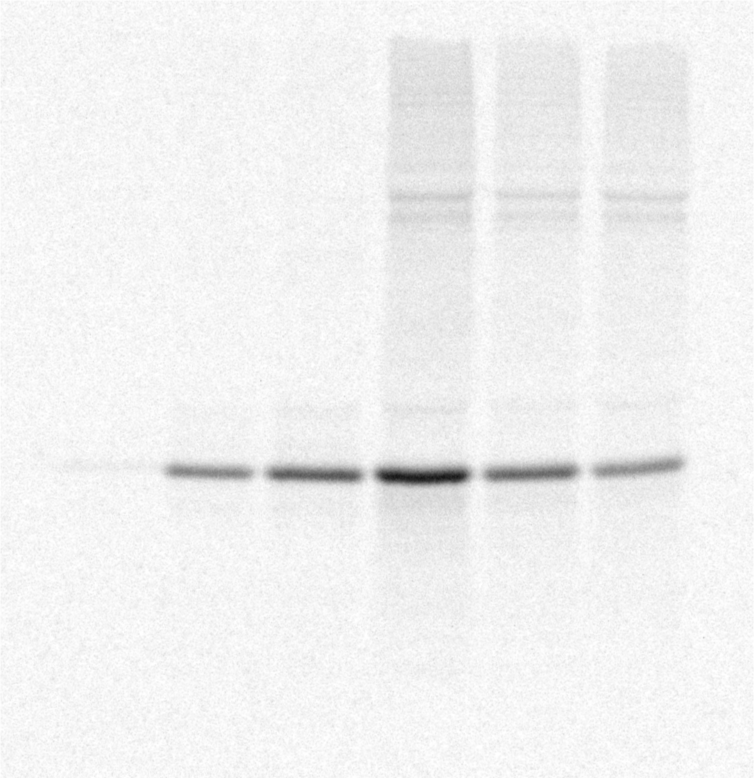

Thapsigargin

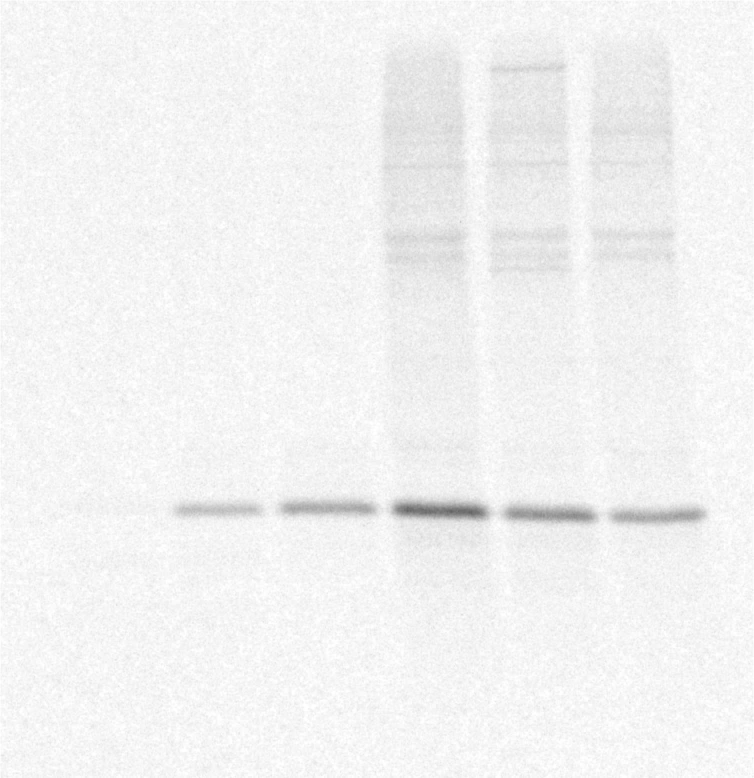

GSK

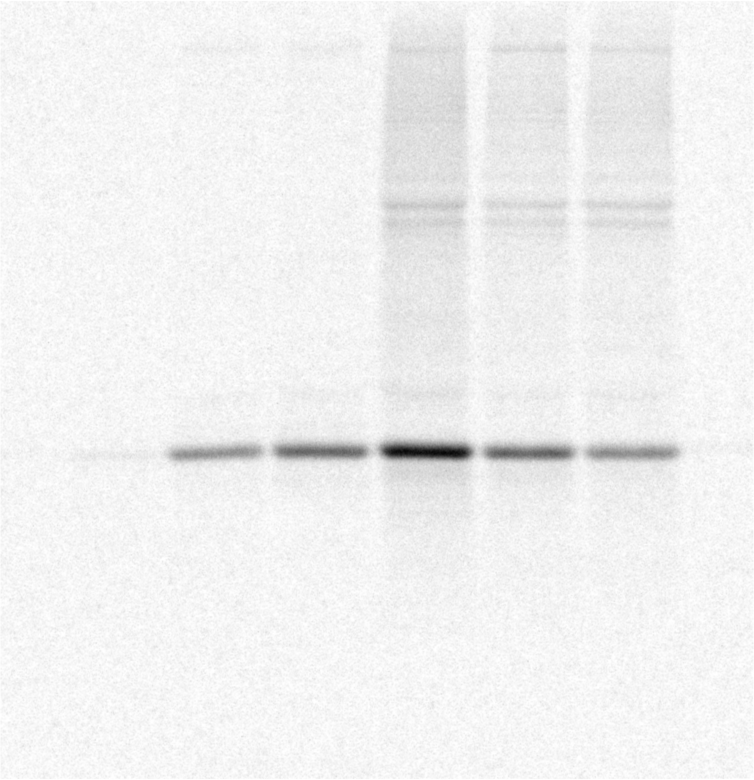

Thapsigargin and GSK

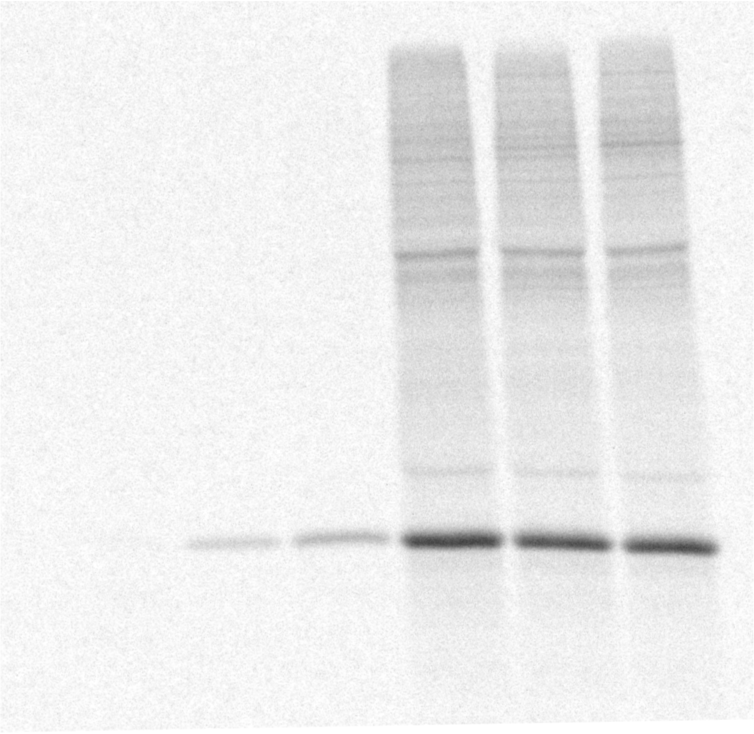

Figure S1F Raw Data (Whole Gels

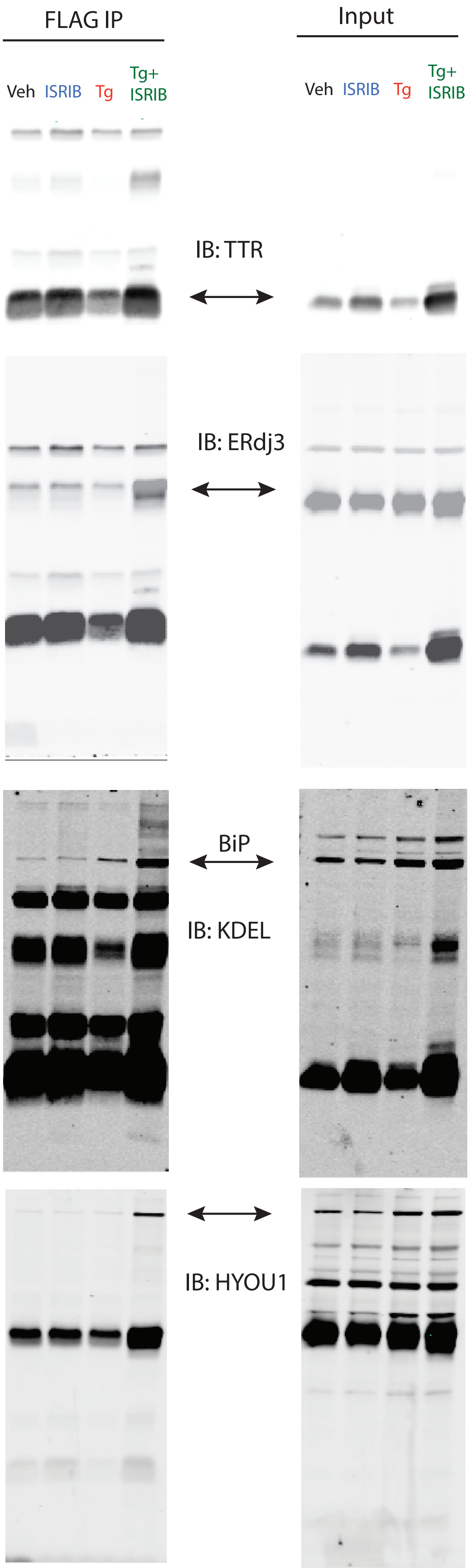

Figure S3 Raw Data (Whole Gels)

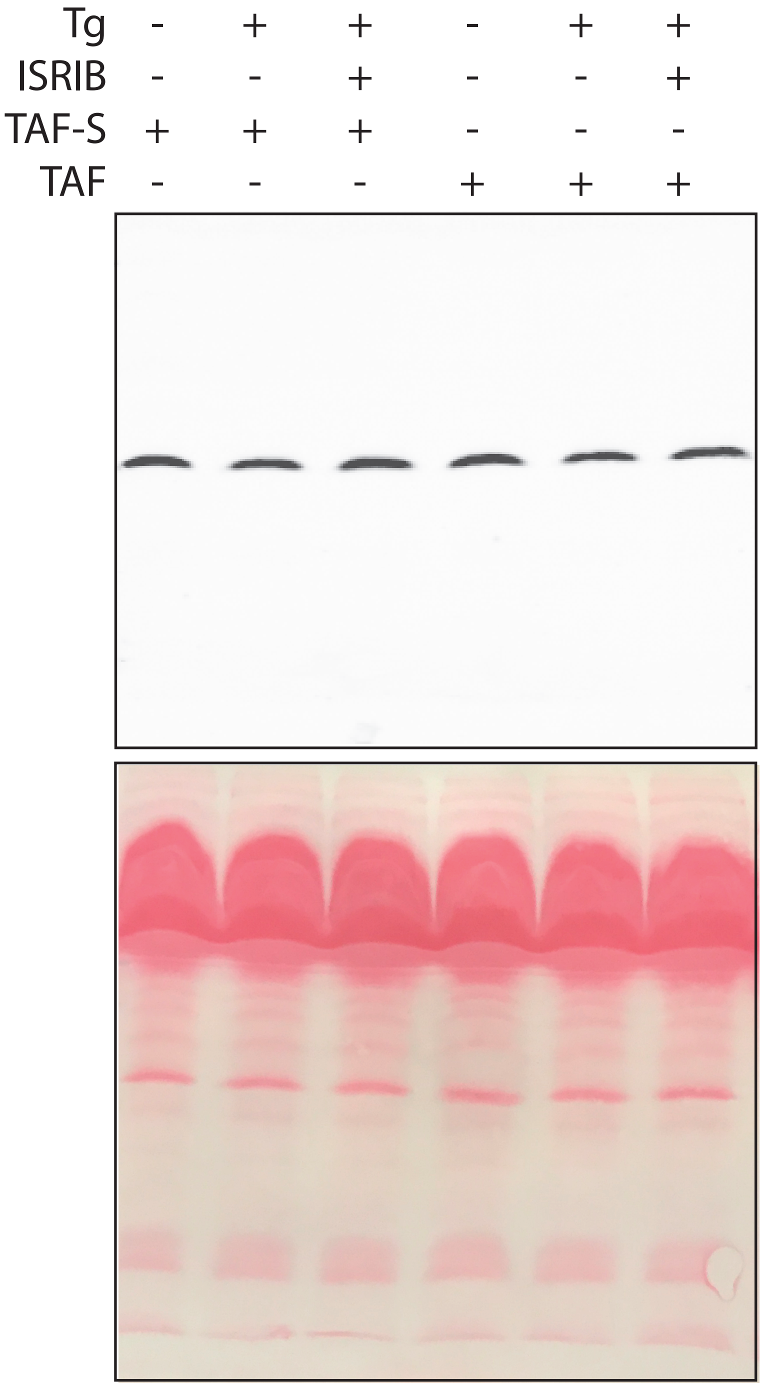

Figure S4A Raw Data (Whole Gels)

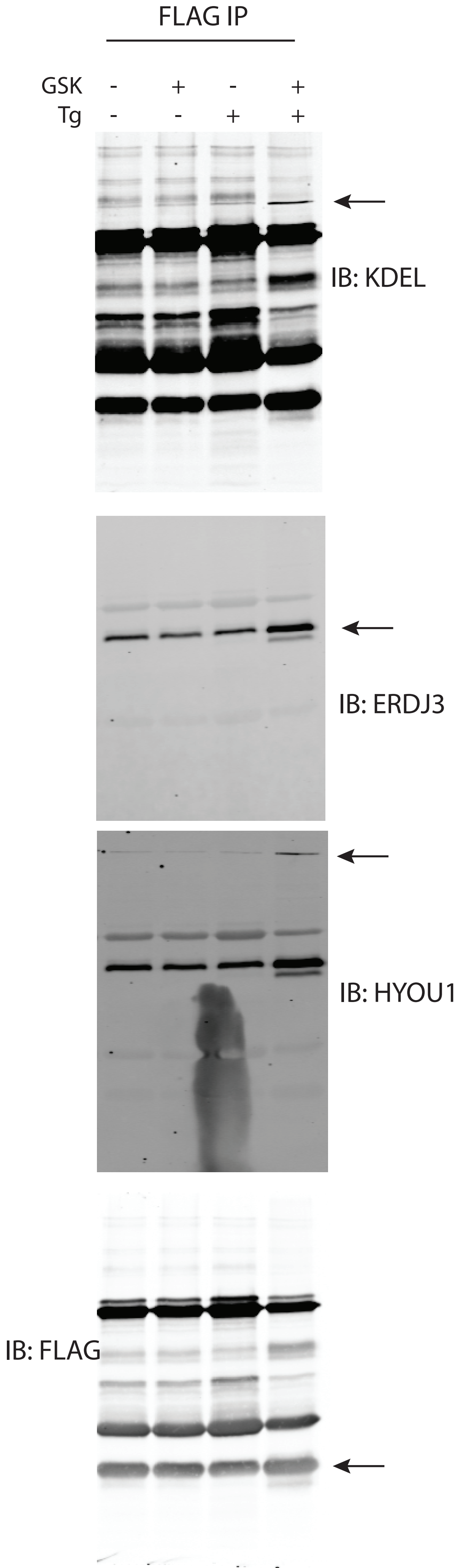

Figure S4B Raw Data (Whole Gels)

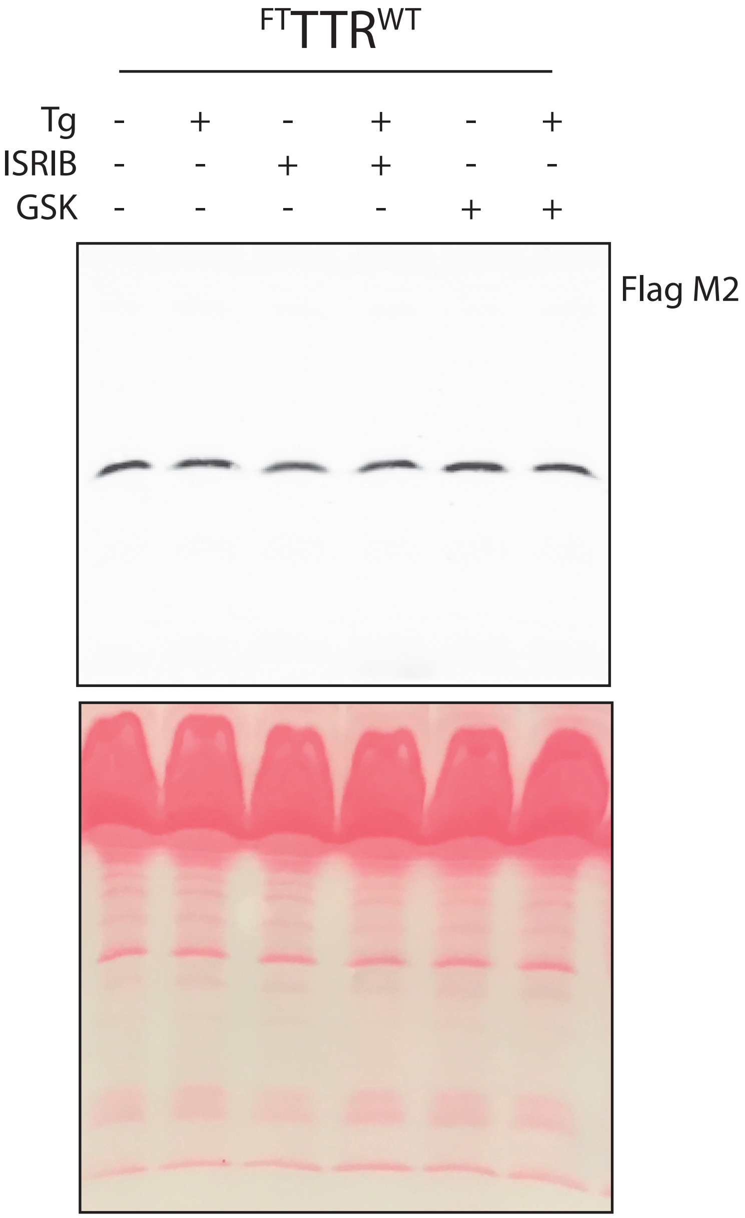

Figure S4C Raw Data (Whole Gels)

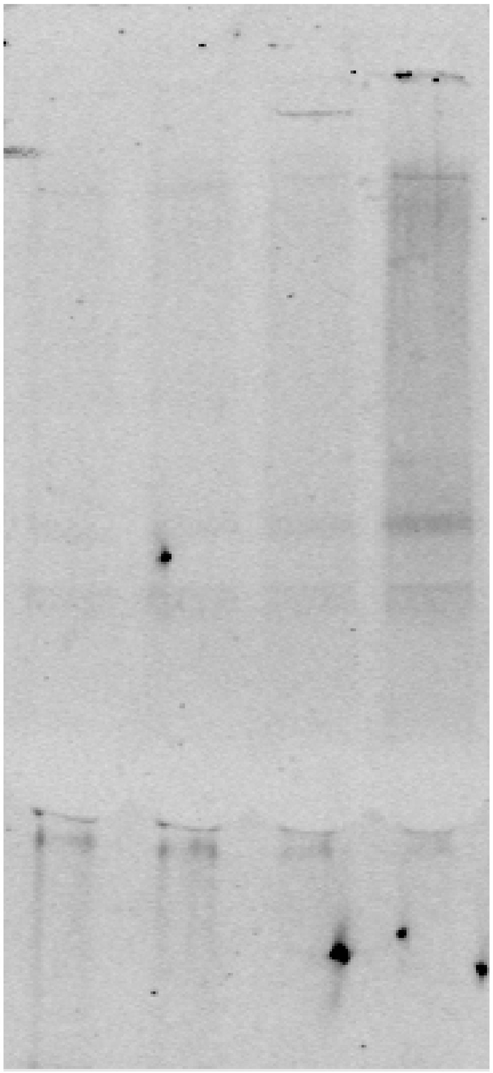

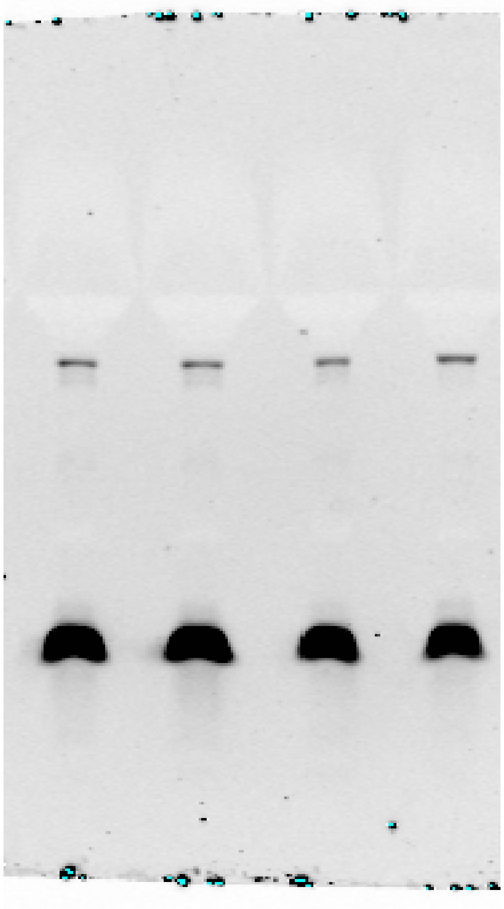

Supplement: Supplementary file 1 — Supplemental Information [file 41598_2018_37207_MOESM1_ESM.pdf]
